# DCLK1 Plays a Metastatic-Promoting Role in Human Breast Cancer Cells

**DOI:** 10.1155/2019/1061979

**Published:** 2019-05-15

**Authors:** Heshu Liu, Tao Wen, Ying Zhou, Xiaona Fan, Tan Du, Tianbo Gao, Lina Li, Jian Liu, Lei Yang, Jiannan Yao, Yang Ge, Guangyu An

**Affiliations:** ^1^Department of Oncology, Beijing Chao-Yang Hospital, Capital Medical University, Beijing 100020, China; ^2^Medical Research Center, Beijing Chao-Yang Hospital, Capital Medical University, Beijing 100020, China

## Abstract

**Background:**

Doublecortin-like kinase 1 (DCLK1) has been universally identified as a cancer stem cell (CSC) marker and is found to be overexpressed in many types of cancers including breast cancer. However, there is little data regarding the functional role of DCLK1 in breast cancer metastasis. In the present study, we sought to investigate whether and how DCLK1 plays a metastatic-promoting role in human breast cancer cells.

**Methods:**

We used Crispr/Cas9 technology to knock out DCLK1 in breast cancer cell line BT474, which basically possesses DCLK1 at a higher level, and stably overexpressed DCLK1 in another breast cancer cell line, T47D, that basically expresses DCLK1 at a lower level. We further analyzed the alterations of metastatic characteristics and the underlying mechanisms in these cells.

**Results:**

It was shown that, compared with the corresponding control cells, DCLK1 overexpression led to an increase in metastatic behaviors including enhanced migration and invasion of T47D cells. By contrast, forced depletion of DCLK1 drastically inhibited these metastatic characteristics in BT474 cells. Mechanistically, the epithelial-mesenchymal transition (EMT) program, which is critical for cancer metastasis, was prominently activated in DCLK1-overexpressing cancer cells, evidenced by a decrease in an epithelial marker ZO-1 and an enhancement in several mesenchymal markers including ZEB1 and Vimentin. In addition, DCLK1 overexpression induced the ERK MAPK pathway, which resultantly enhanced the expression of MT1-MMP that is also involved in cancer metastasis. Knockout of DCLK1 could reverse these events, further supporting a metastatic-promoting role for DCLK1.

**Conclusions:**

Collectively, our data suggested that DCLK1 overexpression may be responsible for the increased metastatic features in breast cancer cells. Targeting DCLK1 may become a therapeutic option for breast cancer metastasis.

## 1. Introduction

Breast cancer (BC) is the most common cancer in women worldwide and the second leading cause among cancer-related deaths in women [1]. In 2017, approximately 252,710 new breast cancer patients and 40,610 cancer-related deaths occurred among US women [2]. In China, the morbidity and mortality of breast cancer have kept rising during past decades, with the mortality-to-incidence ratio increasing [3, 4]. Cancer metastasis is the main culprit of death in breast cancer patients, and patients with metastasis exhibit a poor 5-year survival rate of as low as 22% [5]. So far, the molecular mechanisms underlying BC progression and metastasis are largely undefined.

Doublecortin-like kinase 1 (DCLK1), a member of the protein kinase super family and the doublecortin family, was initially recognized as one type of microtubule-associated proteins (MAPs) involved in neurogenesis and neuronal migration [6]. Recently, DCLK1 has been identified as a cancer stem cell (CSC) marker [7, 8] and was overexpressed in many types of cancer, including colorectal cancer, pancreatic cancer, esophageal cancer, renal clear cell carcinoma (RCC), and breast cancer [9–14]. There is growing evidence indicating that DCLK1 is essential for maintaining cancer stemness and promotion of cancer initiation and metastasis [7, 15, 16]. Upregulation of DCLK1 is clinically associated with the aggressiveness and poor prognosis of several cancers, whereas targeting DCLK1 via a specific monoclonal antibody can block cancer cell invasion and metastasis, thus suggestive of a potential therapeutic target for DCLK1 in cancer metastasis [15, 17]. Available evidence has shown that DCLK1 is overexpressed in breast cancer tissues and related to disease progression and survival [14]; however, it is unclear whether and how DCLK1 plays a decisive role in breast cancer metastasis.

Here, we used overexpression and CRISP/Cas9-mediated knockout of DCLK1 to study its metastatic-promoting role in breast cancer cells and demonstrated that DCLK1 overexpression significantly enhanced metastatic features whereas knockout of DCLK1 could inhibit breast cancer cell migration and invasion. Mechanistically, we found that activation of the EMT program and ERK MAPK pathway may jointly contribute to DCLK1-mediated cancer metastasis.

## 2. Materials and Methods

### 2.1. Cell Culture

The human breast cancer cell lines (BT474, T47D) and HEK293T cells were obtained from Cancer Hospital of the Chinese Academy of Medical Sciences (Beijing, China). The BT474 and T47D cell lines were cultured in RPMI-1640 medium (Sigma, USA) supplemented with 10% fetal bovine serum (FBS). HEK293T cells were cultured in DMEM (Sigma, USA) with 10% FBS. All the cells were cultured in a humidified incubator with 5% CO_2_ at 37°C.

### 2.2. RNA Extraction and Semiquantitative Real-Time PCR

Total RNA from breast cancer cells was extracted by TRIzol reagent (Invitrogen, Life Technologies, Carlsbad, CA) according to the manufacturer's instruction. Then 1 *μ*g total RNA was used for cDNA synthesis in a 20 *μ*l reverse transcription reaction using PrimeScript (Takara, Dalian, China). For the detection of the DCLK1 mRNA expression, real-time PCR analysis was carried out on the 7500 Sequence Detection System (Applied Biosystems, China) using SYBR Green Premix (Invitrogen, USA) according to the manufacturer's instructions. The sequences of the primers were as follows: DCLK1, forward primer: 5′-CGGTCCACATGCAATAAA AA-3′, reverse primer: 5′-GATATCACCGATGCCATCAAG-3′; GAPDH, forward primer: 5′-AATCCCATCACCATCTTCCA-3′, reverse primer: 5′-TGGACTCCACGACGTACTCA-3′. All experiments were performed in triplicate. The relative expression levels of DCLK1 mRNA were determined by 2−ΔΔCT method after normalization to GAPDH.

### 2.3. Protein Isolation and Western Blot

Total protein lysates were composed of RIPA lysis buffer (Beyotime Biotechnology, Shanghai, China) supplemented with 1 mM protease inhibitor cocktail (Beyotime) and 1 mM phenylmethylsulfonyl fluoride (PMSF, Beyotime). The concentration of total protein was measured using BCA Protein Assay Kit (Thermo Fisher Scientific). Protein from each sample was denatured after being boiled for 10 min at 95°C. Then equal amounts of proteins were separated on a 10% SDS polyacrylamide gel and transferred onto a PVDF membrane (Millipore Corporation, Billerica, MA, USA). The membrane was blocked using 5% nonfat milk (BD, 232100, USA) for 1 hour at room temperature and probed overnight at 4°C with specific primary antibodies. After being washed three times with TBST, membranes were subsequently incubated with secondary antibodies (1:8000, ZSGB-BIO, China) for 1 hour at room temperature. Signals were detected using the Bio-Rad imaging system (Bio-Rad ChemiDoc MP, 1708195; Bio-Rad Laboratories, Inc., Hercules, CA, USA) using chemiluminescent HRP substrate (EMD Millipore). The antibodies used above were anti-DCLK1 (1:1000, Abcam), anti-ZEB1 (1:1000, Cell Signaling Technology), anti-Vimentin (1:1000, Cell Signaling Technology), anti-MT1-MMP (1:500, Cell Signaling Technology), anti-ZO-1 (1:1000, Cell Signaling Technology), anti-ERK1/2 (1:1000, Cell Signaling Technology), and anti-phospho-ERK1/2 (1:1000, Cell Signaling Technology).

### 2.4. Knockout and Overexpression of DCLK1

One pair of single guide RNA (sgRNA) was designed specifically targeting DCLK1. The sgRNA sequence is as follows: Oligo1: 5′-CACCGGAGTAGAGAGCTGA CTACCA-3′, Oligo2: 5′-AAACTGGTAGTCAGCTCTCTACTCC-3′. The sgRNA sequence was cloned into predigested lentiCRISPRv2 plasmids, and then the reconstructive vector was transformed into Stbl3 growing on LB with Ampicillin. After being confirmed by sequencing, the expression plasmids constructed above were cotransfected along with packaging plasmids pMD2G and psPAX2 into HEK293T cells using lipofectamine 3000 (Invitrogen, USA). The lentivirus targeting DCLK1 gene in Crispr/Cas9 system was harvested at 48 h and 72 h posttransfection and transfected into BT474 cells in a 6-well plate (Corning) with polybrene. These infected cells were selected for 7 days with 1.5 *μ*g/ml puromycin (Gibco, USA) to establish stable cell lines. The DCLK1 overexpression plasmid (pCDH-DCLK1-GFP) was purchased from Beijing AUGCT (China). Lentivirus packaging and methods of infecting cells were as described above.

### 2.5. Transwell Migration and Invasion Assay

The transwell assay was performed in a 24-well chamber (Corning Incorporated, Corning, NY, USA), which was precoated with or without Matrigel (BD Bioscience, USA). 2 × 10^5^ cells with FBS-free medium were plated into the upper chamber. Cell culture medium containing 10% FBS was added to the bottom of each well as chemoattractant. After being cultured for 24 h, noninvaded or nonmigrating cells were removed off the upper chamber using a cotton swab while the remaining cells were fixed with 4% paraformaldehyde for 10 min and stained with 0.1% crystal violet for 5 min. After drying, the migrating cells under 5 random views were counted.

### 2.6. Statistical Analysis

Data analysis was performed by GraphPad Prism 7. Differences were analyzed by Student's t-test (unpaired, 2-tailed) and p<0.05 was considered statistically significant. Independent experiments were repeated at least in triplicate and quantitative data were exhibited as mean ± standard deviation (SD).

## 3. Results

### 3.1. Detection of Basic Expression of DCLK1 in Breast Cancer Cell Lines

**Figure 1:**
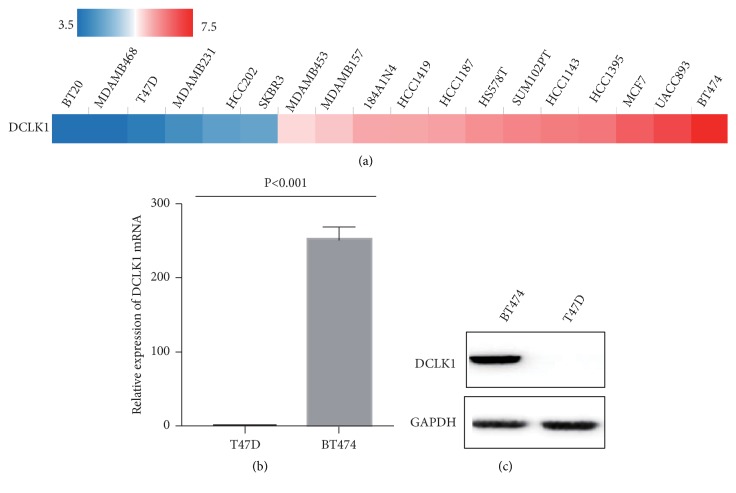
*Detection of basic expression of DCLK1 in breast cancer cell lines*. (a) The expression of DCLK1 in breast cancer cell lines (TCGA). (b) Real-time PCR analysis of the relative mRNA levels of DCLK1 in 2 BC cell lines (BT474, T47D). (c) Western blotting analysis of the relative protein levels of DCLK1 in 2 BC cell lines.

We first analyzed basic expression levels of DCLK1 in breast cancer cell lines by TCGA (Breast Cancer Cell Lines) (Figure 1(a)). We then chose two breast cancer cell lines (BT474, T47D) according to TCGA database and performed qRT-PCR and western blot to evaluate their expression of DCLK1. The results showed that DCLK1 is expressed relatively higher in BT474 cell line and relatively lower in T47D cell line at its mRNA and protein levels (Figures 1(b) and 1(c)).

### 3.2. DCLK1 Promotes Breast Cancer Cell Migration and Invasion

**Figure 2:**
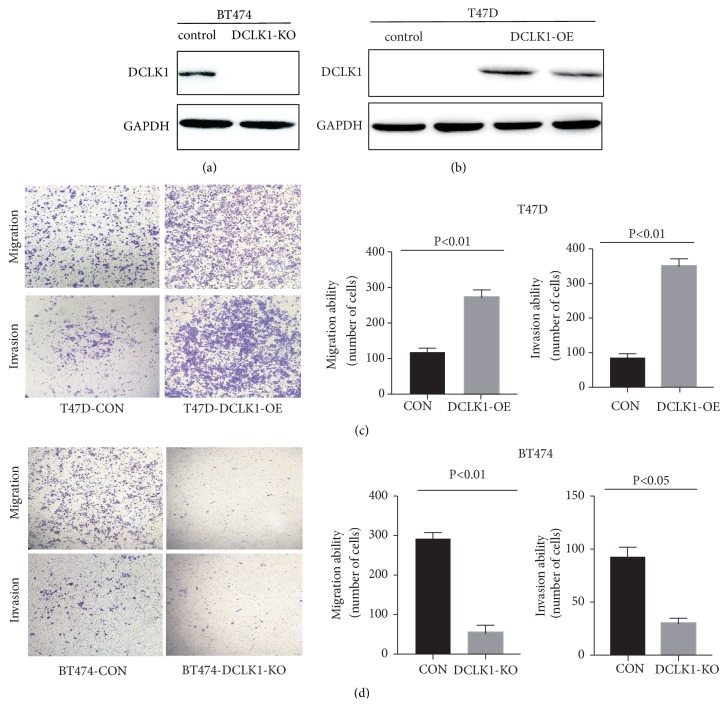
*DCLK1 promotes breast cancer cells migration and invasion*. (a) CRISPR/Cas9-mediated DCLK1 knockout was confirmed by western blot. (b) DCLK1 overexpression in T47D cells was confirmed by western blot. (c) Transwell migration and invasion assays in T47D cells overexpressing DCLK1 and their corresponding control cells. (d) Transwell analysis on the effect of DCLK1 deletion on cell migratory and invasive abilities of BT474 cell line. Results are exhibited as mean ± SD.

To investigate the functional role of DCLK1, we used a precise gene editing (CRISPR/Cas9) technology to knock out DCLK1 in BT474 cells with specific sgRNAs targeting DCLK1. Knockout of DCLK1 was confirmed by western blot (Figure 2(a)). In the meanwhile, another breast cancer cell line T47D was stably transfected with pCDH-DCLK1-GFP (containing DCLK1 sequences). Overexpression of DCLK1 in T47D cells was also confirmed by western blot (Figure 2(b)). We then performed transwell assays to investigate cell migratory and invasive abilities. The results showed that DCLK1 overexpression markedly increased the number of migrating and invaded cells, respectively, as compared with the corresponding control cells (Figure 2(c)). By contrast, DCLK1 deletion drastically inhibited both migratory and invasive abilities in BT474-KO cells (Figure 2(d)). Overall, these results indicated that DCLK1 may promote metastatic abilities of breast cancer cells.

### 3.3. DCLK1 Activates the Epithelial‐Mesenchymal Transition (EMT) Process

**Figure 3:**
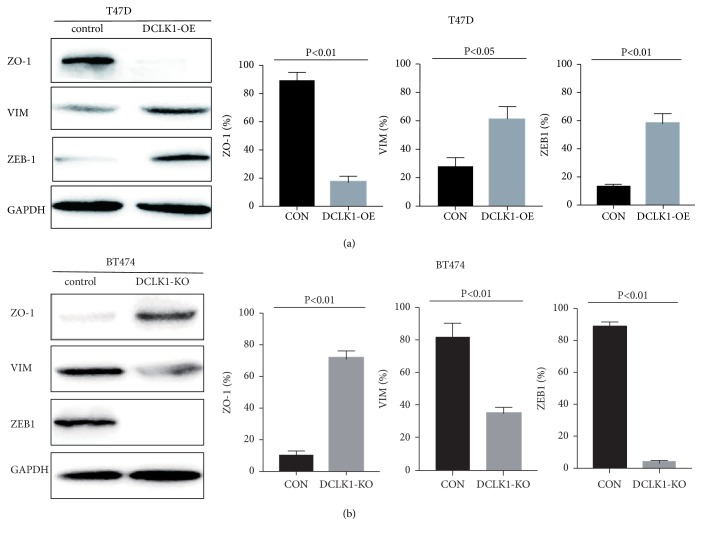
*DCLK1 overexpression/knockout induces phenotypic changes of EMT markers*. (a) Western blot analysis of the effect of DCLK1 overexpression on EMT markers at protein levels. (b) Western blot analysis of the influence of DCLK1 knockout on EMT markers at protein levels. Experiments were performed at least in triplicate and representative images were shown.

We next sought to explore the mechanisms underlying DCLK1-mediated metastatic alterations in breast cancer cells. Considering that the EMT and MAPK pathways are obvious candidates involved in cancer progression and metastasis in many cancer types, we checked whether DCLK1 affects these signaling pathways. The results demonstrated that DCLK1 overexpression led to significantly increased characteristics of the EMT process in T47D cells. The expression of an epithelial marker ZO-1 was remarkably decreased in DCLK1-overexpressing cells compared to the control cells. Accordingly, the expression of Vimentin and ZEB1, which are the mesenchymal markers, was upregulated following DCLK1 overexpression in T47D cells (Figure 3(a)). Concurrently, knockout of DCLK1 in BT474 cells profoundly reduced the EMT characteristics, evidenced by an increase in the expression of ZO-1, accompanied by a decrease in the expression of Vimentin and ZEB1 (Figure 3(b)).

### 3.4. DCLK1 Activates ERK MAPK Pathway in Breast Cancer Cells

**Figure 4:**
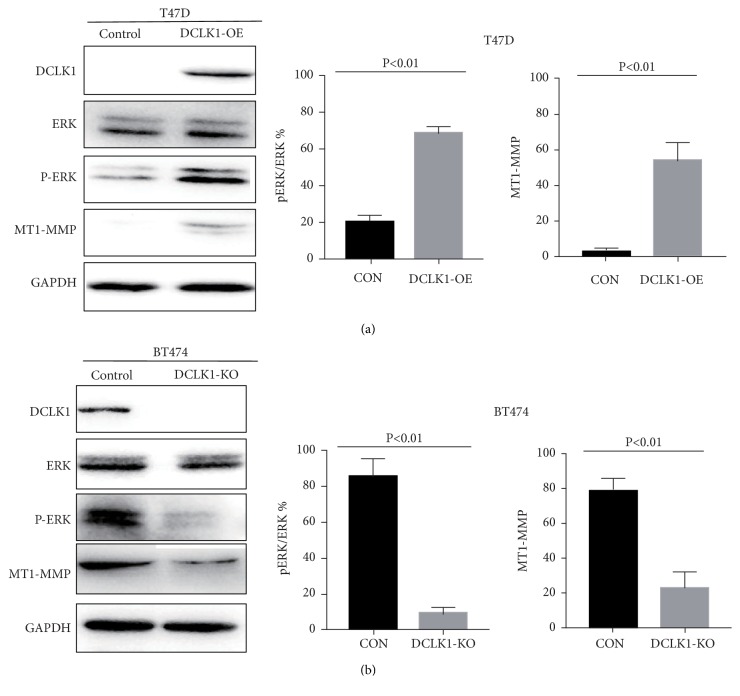
*DCLK1 activates ERK signaling pathway in breast cancer cells*. Western blot analysis of the effect of DCLK1 on ERK MAPK signaling pathway and downstream molecule MT1-MMP in DCLK1-OE cells (a) and DCLK1-KO cells (b). Experiments were performed at least in triplicate and representative images were shown.

There is growing evidence for a significant role of MAPK signaling pathway involved in cancer metastasis [18, 19]. Here we found that DCLK1 overexpression induced the phosphorylation of ERK MAPK pathway (Figure 4(a)), but not JNK or p38 MAPK pathways, in T47D cells. Previous studies demonstrated that activation of ERK MAPK pathway can promote the expression of MT1-MMP, which is a key protease and plays an important role in pro-migration proteolysis and is thereby involved in many cancer metastases [20–22]. We observed that DCLK1 overexpression caused an upregulation of MT1-MMP, which was likely due to activation of ERK MAPK pathway (Figure 4(a)). Likewise, knockout of DCLK1 in BT474 cells inhibited the phosphorylation of ERK MAPK pathway as well as the expression of MT1-MMP (Figure 4(b)). Collectively, these results showed that activation of EMT and ERK MAPK pathway may be possibly responsible for DCLK1-mediated metastatic-promoting effects. DCLK1 may act as a molecular target in the treatment of breast cancer metastasis.

## 4. Discussion

Cancer metastases are the main reasons of cancer-related deaths, especially for breast cancer, whose metastasis accounts for over 90% of cancer-related deaths worldwide [23]. Although patients with breast cancers have undertaken effective excision or radio (chemo) therapy, their survival rates would significantly decrease once distant metastasis occurs. Therefore, key molecular markers indicating metastasis in breast cancer need to be explored and identified urgently.

Accumulating evidence has suggested that cancer stem cells (CSCs) may play a pivotal role in tumor initiation, promotion, and metastasis for their self-renewal and differentiation ability [24]. Recent studies suggest that DCLK1, a newly identified cancer stem cell marker, can mark gastrointestinal cancer stem cells and is closely associated with cancer metastasis and angiogenesis in many cancers [25, 26]. For example, Gao et al. reported that DCLK1 was upregulated in human colorectal cancer and correlated with metastasis and advanced TNM stage [9]. Weygant et al. showed that DCLK1 was overexpressed in stage II-III RCCs, compared with normal kidney and stage I tumors [12], and could predict survival and recurrence of RCC [15]. And in pancreatic cancer, DCLK1 also played an important role in promoting metastasis and predicting survival [11]. As for breast cancer, Wang et al. demonstrated that the expression of DCLK1 was significantly increased in basal-like breast cancer tissues compared with normal mammary tissues, and DCLK1 overexpression could predict poor prognosis [27]. However, Liu et al. found that DCLK1 was positively related to favorable clinic-pathologic features and might be a good prognostic factor in breast cancer, especially in invasive breast cancers with neuroendocrine differentiation [28]. These contradicting reports indicated that the precise role of DCLK1 in breast cancer remains to be further elucidated. Moreover, whether DCLK1 contributes to breast cancer metastasis is still unclear. In this study, we used a precise gene editing to knock out DCLK1 as well as overexpression of DCLK1 in two types of breast cancer cells to investigate its functional role. We found that DCLK1 promoted cell migratory and invasive abilities, which are typical characteristics of cancer metastasis. Our findings confirmed a metastatic-promoting role of DCLK1 in breast cancer, which was consistent with the role of DCLK1 in many other cancers.

We next explored how DCLK1 affects metastatic ability of breast cancer cells. It is known that EMT plays a key role in cancer progression to metastasis [29–31]. Stationary epithelial tumor cells undergo EMT to acquire mesenchymal phenotypes and motility and invasion abilities [32]. We found that DCLK1 overexpression activated the EMT process, demonstrated by a reduced expression of epithelial cell marker, such as ZO-1, and an increased expression of mesenchymal marker, such as Vimentin and ZEB1, although E-cad was not detectable in cells. In addition, MAPK signaling pathways are also involved in cancer malignancies such as proliferation, invasion, and metastasis. We thereby determined whether DCLK1 has an influence on MAPK pathway. Here, we only observed that ERK MAPK pathway, but not JNK or p38 MAPK pathways, was activated in DCLK1-overexpressing cells. Knockout of DCLK1 could block the phosphorylation of ERK MAPK pathway.

The ability of cancer cells to degrade the extracellular matrix (ECM) is also essential for cancer metastasis, which requires the involvement of matrix metalloproteinases (MMPs). Of these, MT1-MMP (membrane type-1 matrix metalloproteinase, MMP14) is a key protease that activates pro-MMPs such as MMP2 and MMP9 and is associated with ECM degradation and cell migration during cancer metastasis [33, 34]. Cepeda et al. reported that optimal levels of active MT1-MMP required the elevated pERK levels in breast cancer [35]. Lin et al. also showed that MT1-MMP mediated breast cancer invasion possibly through activating MAPK/ERK pathway [36]. Our data showed that there was an association between DCLK1 and MT1-MMP, supporting the likelihood that DCLK1 upregulates the expression of MT1-MMP via activation of the MAPK/ERK pathway.

In summary, we showed that DCLK1 activates the EMT program and promotes the MAPK/ERK pathway to elevate expression of MT1-MMP, which collectively contributes to breast cancer metastasis. DCLK1 may be considered as a promising therapeutic target to block metastasis in breast cancer.

## Data Availability

The data used to support the findings of this study are available from the corresponding author upon request.

## References

[1] Siegel R. L., Miller K. D., Jemal A. (2018). Cancer statistics, 2018. *CA: A Cancer Journal for Clinicians*.

[2] DeSantis C. E., Ma J., Goding Sauer A., Newman L. A., Jemal A. (2017). Breast cancer statistics, 2017, racial disparity in mortality by state. *CA: A Cancer Journal for Clinicians*.

[3] Cheng Y., Yan Y., Gong J., Yang N., Nie S. (2018). Trends in incidence and mortality of female breast cancer during transition in Central China. *Cancer Management and Research*.

[4] Sung H., Rosenberg P. S., Chen W.-Q. (2015). Female breast cancer incidence among Asian and western populations: more similar than expected. *Journal of the National Cancer Institute*.

[5] Cheng Y. C., Ueno N. T. (2012). Improvement of survival and prospect of cure in patients with metastatic breast cancer. *Breast Cancer*.

[6] Lin P. T., Gleeson J. G., Corbo J. C., Flanagan L., Walsh C. A. (2000). DCAMKL1 encodes a protein kinase with homology to doublecortin that regulates microtubule polymerization. *The Journal of Neuroscience*.

[7] Nakanishi Y., Seno H., Fukuoka A. (2013). Dclk1 distinguishes between tumor and normal stem cells in the intestine. *Nature Genetics*.

[8] Bailey J. M., Alsina J., Rasheed Z. A. (2014). DCLK1 marks a morphologically distinct subpopulation of cells with stem cell properties in preinvasive pancreatic cancer. *Gastroenterology*.

[9] Gao T., Wang M., Xu L., Wen T., Liu J., An G. (2016). DCLK1 is up-regulated and associated with metastasis and prognosis in colorectal cancer. *Journal of Cancer Research and Clinical Oncology*.

[10] Zhou B., Sun C., Hu X. (2018). MicroRNA-195 Suppresses the Progression of Pancreatic Cancer by Targeting DCLK1. *Cellular Physiology and Biochemistry*.

[11] Li J., Wang Y., Ge J. (2018). Doublecortin-like kinase 1 (DCLK1) regulates B cell-specific moloney murine leukemia virus insertion site 1 (Bmi-1) and is associated with metastasis and prognosis in pancreatic cancer. *Cellular Physiology and Biochemistry*.

[12] Weygant N., Qu D., May R. (2015). DCLK1 is a broadly dysregulated target against epithelial-mesenchymal transition, focal adhesion, and stemness in clear cell renal carcinoma. *Oncotarget *.

[13] Whorton J., Sureban S. M., May R. (2015). DCLK1 is detectable in plasma of patients with Barrett's esophagus and esophageal adenocarcinoma. *Digestive Diseases and Sciences*.

[14] Lv Y., Song G., Wang R., Di L., Wang J. (2017). Doublecortin-like kinase 1 is a novel biomarker for prognosis and regulates growth and metastasis in basal-like breast cancer. *Biomedicine & Pharmacotherapy*.

[15] Ge Y., Weygant N., Qu D. (2018). Alternative splice variants of DCLK1 mark cancer stem cells, promote self-renewal and drug-resistance, and can be targeted to inhibit tumorigenesis in kidney cancer. *International Journal of Cancer*.

[16] Chandrakesan P., Yao J., Qu D. (2017). Dclk1, a tumor stem cell marker, regulates pro-survival signaling and self-renewal of intestinal tumor cells. *Molecular Cancer*.

[17] Weygant N., Qu D., Berry W. L. (2014). Small molecule kinase inhibitor LRRK2-IN-1 demonstrates potent activity against colorectal and pancreatic cancer through inhibition of doublecortin-like kinase 1. *Molecular Cancer*.

[18] Lu H., Guo Y., Gupta G., Tian X. (2019). Mitogen-activated protein kinase (MAPK): new insights in breast cancer. *Journal of Environmental Pathology, Toxicology and Oncology*.

[19] Lemaître C., Tsang J., Bireau C., Heidmann T., Dewannieux M., Ross S. R. (2017). A human endogenous retrovirus-derived gene that can contribute to oncogenesis by activating the ERK pathway and inducing migration and invasion. *PLoS Pathogens*.

[20] Wang X., Zhao X., Yi Z. (2018). WNT5A promotes migration and invasion of human osteosarcoma cells via SRC/ERK/MMP-14 pathway. *Cell Biology International*.

[21] Wang C., Li Z., Shao F. (2017). High expression of Collagen Triple Helix Repeat Containing 1 (CTHRC1) facilitates progression of oesophageal squamous cell carcinoma through MAPK/MEK/ERK/FRA-1 activation. *Journal of Experimental & Clinical Cancer Research*.

[22] Cepeda M. A., Evered C. L., Pelling J. J. H., Damjanovski S. (2017). Inhibition of MT1-MMP proteolytic function and ERK1/2 signalling influences cell migration and invasion through changes in MMP-2 and MMP-9 levels. *Journal of Cell Communication and Signaling*.

[23] Wang Y., Zhou B. P. (2011). Epithelial-mesenchymal transition in breast cancer progression and metastasis. *Chinese Journal of Cancer*.

[24] Ayob A. Z., Ramasamy T. S. (2018). Cancer stem cells as key drivers of tumour progression. *Journal of Biomedical Science*.

[25] Park S., Kim J., Choi J. (2019). Inhibition of LEF1-mediated DCLK1 by niclosamide attenuates colorectal cancer stemness. *Clinical Cancer Research*.

[26] Ikezono Y., Koga H., Akiba J. (2017). Pancreatic neuroendocrine tumors and EMT behavior are driven by the CSC marker DCLK1. *Molecular Cancer Research*.

[27] Wang J., Wang S., Zhou J., Qian Q. (2018). miR-424-5p regulates cell proliferation, migration and invasion by targeting doublecortin-like kinase 1 in basal-like breast cancer. *Biomedicine & Pharmacotherapy*.

[28] Liu Y., Tsang J. Y., Ni Y. (2016). Doublecortin-like kinase 1 expression associates with breast cancer with neuroendocrine differentiation. *Oncotarget*.

[29] Yeung K. T., Yang J. (2017). Epithelial-mesenchymal transition in tumor metastasis. *Molecular Oncology*.

[30] Pastushenko I., Brisebarre A., Sifrim A. (2018). Identification of the tumour transition states occurring during EMT. *Nature*.

[31] Pastushenko I., Blanpain C. (2019). EMT transition states during tumor progression and metastasis. *Trends in Cell Biology*.

[32] Nieto M. A. (2013). Epithelial plasticity: a common theme in embryonic and cancer cells. *Science*.

[33] Zarrabi K., Dufour A., Li J. (2011). Inhibition of Matrix Metalloproteinase 14 (MMP-14)-mediated cancer cell migration. *The Journal of Biological Chemistry*.

[34] Sakamoto T., Seiki M. (2017). Integrated functions of membrane-type 1 matrix metalloproteinase in regulating cancer malignancy: beyond a proteinase. *Cancer Science*.

[35] Cepeda M. A., Pelling J. J., Evered C. L. (2016). Less is more: low expression of MT1-MMP is optimal to promote migration and tumourigenesis of breast cancer cells. *Molecular Cancer*.

[36] Lin Y., Chang G., Wang J. (2011). NHE1 mediates MDA-MB-231 cells invasion through the regulation of MT1-MMP. *Experimental Cell Research*.

